# Electrospun Polycaprolactone Membranes Expanded with Chitosan Granules for Cell Infiltration

**DOI:** 10.3390/polym16040527

**Published:** 2024-02-15

**Authors:** Tânia Vieira, Ana Margarida Rebelo, João Paulo Borges, Célia Henriques, Jorge Carvalho Silva

**Affiliations:** 1 Centro de Investigação de Materiais, Institute for Nanostructures, Nanomodelling and Nanofabrication, CENIMAT-I3N, 2829-516 Caparica, Portugal; jpb@fct.unl.pt (J.P.B.); crh@fct.unl.pt (C.H.); 2 Departamento de Física, Faculdade de Ciências e Tecnologia, Universidade Nova de Lisboa, 2829-516 Caparica, Portugal; 3 Departamento de Ciência dos Materiais, Faculdade de Ciências e Tecnologia, Universidade Nova de Lisboa, 2829-516 Caparica, Portugal

**Keywords:** electrospinning, polycaprolactone, chitosan, pores, cell infiltration

## Abstract

The small pore size of electrospun membranes prevents their use as three-dimensional scaffolds. In this work, we produced polycaprolactone (PCL) electrospun fibrous membranes with expanded pores by incorporating chitosan (CS) granules into the PCL solution. Scanning electron microscopy images confirmed the presence of the CS granules embedded in the PCL fibers, creating an open structure. Tensile testing results showed that the addition of CS decreased both Young’s modulus and the yield stress, but co-electrospun membranes (PCL fibers blended with CS-containing PCL fibers) exhibited higher values compared to single electrospun membranes (CS-containing PCL fibers). Human fibroblasts adhered to and proliferated on all scaffolds. Nuclear staining revealed that cells populated the entire scaffold when CS granules were present, while in PCL membranes, cells were mostly limited to the surface due to the small pore size. Overall, our findings demonstrate that electrospun membranes containing CS granules have sufficiently large pores to facilitate fibroblast infiltration without compromising the mechanical stability of the structure.

## 1. Introduction

Regenerative medicine aims at restoring full tissue or organ structure and function, combining cells, scaffolds, and signaling molecules in synergy [1]. Several methods are used to produce 3D scaffolds that resemble the biological and functional organization of tissues, including electrospinning, 3D printing, and freeze-drying [2]. Electrospinning is a simple and versatile technique that allows the production of fibrous membranes with diameters ranging from tens of nanometers to a few micrometers, which mimic the structural characteristics of the extracellular matrix (ECM) [3]. Fibers produced using the electrospinning technique have high surface area-to-volume ratio, porosity, and pore interconnectivity. The major drawback of electrospun membranes is the small pore size that typically lies in the range of 200 nm to 2 µm [4]. This prevents cellular infiltration, and fibrous membranes behave essentially as 2D structures with a corrugated surface to which cells adhere but are not able to infiltrate, limiting their presence to the scaffold’s surface [5]. Depending on cell type, the scaffolds must have pore sizes varying from 20 µm to 300 µm to allow their infiltration, thereby populating the entire scaffold [6,7,8]. Therefore, porosity with interconnectivity is needed, and standard electrospinning fails to produce such fibrous membranes.

Due to the widespread use of electrospun membranes in tissue engineering, there is a significant need to increase the pore size of the scaffolds, creating 3D structures able to promote cell infiltration [8,9]. Electrospun membranes with increased pore size enhance cell infiltration throughout the scaffold, which is associated with increased vascularization [10,11]. Different methods have been employed to pursue that goal. Those methods include combining electrospinning with salt leaching or gas foaming, cryogenic electrospinning, sacrificial fibers, the combination of nanofibers and microfibers, ultrasonication, electrospinning using a liquid bath collector, and electrospinning with electrospray and custom-made collectors, all of which are described in detail in several reviews [12,13,14,15,16]. 

Water-soluble porogens (e.g., salt or sugar in crystal form) or polymers electrospun or electro-sprayed along with the structural polymers were studied in the expansion of the network of electrospun fibers to create pores and channels that might support cell migration into the scaffold. Polycaprolactone (PCL)/gelatin fibers were produced concurrently with the deposition of electro-sprayed poly(ethylene glycol) (PEG) [17]. After PEG removal, a fibrous scaffold with enlarged pore size was obtained. The scaffolds’ pore size and interconnectivity facilitated cell infiltration throughout [17]. PCL and polyvinylpyrrolidone (PVP) solutions were co-electrospun, and the resulting membranes were placed in water for PVP dissolution. This increased the pore size of the fibrous structure and allowed human dermal fibroblasts to infiltrate the scaffold [18]. 

A different approach lies in the incorporation of microparticles in the fibrous network. Combining electrospun fibers with microparticles improved cellular infiltration when compared to the combination of electrospinning with leaching methods. PCL/collagen electrospun membranes with micron-sized fibers co-electro-sprayed with a hyaluronic acid-derivative hydrogel promoted better cell penetration when compared to membranes co-electrospun with poly(ethylene oxide) (PEO) or gelatin that were leached out [19]. An identical result was obtained by Hodge et al. who produced poly(lactic-co-glycolic acid) (PLGA) scaffolds that were either co-electrospun with PEO microfibers or co-electro-sprayed with PEO microparticles [20]. After the dissolution of PEO, the PLGA scaffolds produced using microparticles displayed increased porous fraction, pore area, and depth of cellular migration through the PLGA scaffold relative to the analogous microfiber scaffolds. Additionally, Zander et al. verified an increase in pore size and cell infiltration on polycaprolactone (PCL) membranes with larger fibers (9 ± 4 µm) than on PCL mats prepared with polyethylene oxide fibers as a sacrificial agent [21]. 

Polycaprolactone (PCL) is a semi-crystalline, biocompatible, and biodegradable polyester with mechanical properties in the range of those of biological tissues [22]. However, PCL is a synthetic polymer lacking biological motifs for cell recognition; therefore, it is usually employed with natural polymers such as chitosan (CS) and gelatin [23]. CS, a natural polysaccharide derived from chitin, is biocompatible, biodegradable, antimicrobial, and hemostatic, making it one of the best studied polymers in the context of tissue engineering [24,25]. CS can be blended with PCL in solution to form fibrous membranes or co-electrospun to combine individual PCL and CS fibers in the membrane [26]. Other methods reported for the combination of CS with PCL involve the adsorption of chitosan nanoparticles onto the PCL nanofibers [27], the coating of PCL/carbon nanotube fibers with CS [28], the covalent grafting of electrospun membranes and 3D-printed PCL scaffolds with CS [29], and the incorporation of CS granules co-electrospun with PCL [30].

The techniques commonly used to increase the pore size of electrospun membranes usually involve additional processing steps or equipment that increase the complexity, time, and final cost of producing the membranes. In this work, we investigated a simple method to expand a nanofiber membrane made of PCL by incorporating CS granules in the fibers: PCL solutions were loaded with CS granules at two different concentrations, 2% and 4%. These solutions were then individually electrospun and co-electrospun to fabricate membranes consisting of PCL fibers and PCL fibers containing the CS granules. The electrospun membranes were characterized regarding their morphology, porosity, mechanical properties, and the effects on cell adhesion, proliferation, and, most importantly, infiltration.

## 2. Materials and Methods

### 2.1. Solution Preparation

Chitosan (Chitopharm S, from Cognis) granules with a characteristic size below 100 µm were prepared using a grinder (MO 3300 from Orbegozo, Murcia, Spain), followed by their passage through a sieve with a pore size of 100 µm using an electromagnetic sieve agitator (AS 300 control from Retsch, Haan, Germany). Solutions for electrospinning were prepared with PCL (Mn = 70–90 kDa, from Sigma-Aldrich, St. Louis, MO, USA) at a concentration of 10 wt% dissolved in chloroform (from Carlo Erba Reagents, Cornaredo, Italy). To this solution, the CS granules, obtained as previously described, were added at 2 wt% or 4 wt% concentrations. Above a concentration of 4 wt% of CS particles, solutions became difficult to electrospin due to the clogging of the needles. These solutions were designated CS2 and CS4, respectively. 

### 2.2. Electrospinning Process

**Figure 1:**
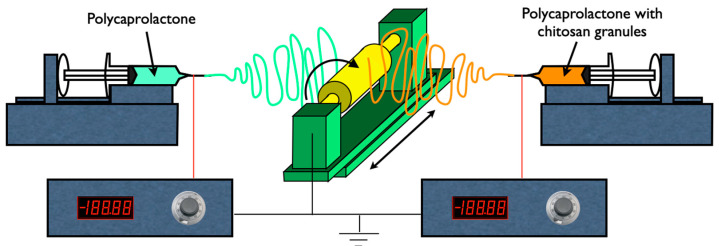
Schematic representation of the electrospinning set-up used in this work. The PCL, CS2, and CS4 membranes are produced using only one syringe pump. The PCS2 and PCS4 membranes are spun using two pumps, one for the PCL solution and the other for the CS2 or CS4 solutions. In both cases, a grounded cylindrical collector with slow rotational and translational movements is used.

Each of the prepared solutions was loaded in 5 mL plastic syringes connected to 20 G (PCL solution) or 18 G (PCL with CS solutions) blunt needles and mounted on a syringe pump (SyringePump NE-300 from New Era Pump Systems, Farmingdale, Nassau County, New York, USA) to establish the flow rate. A high-voltage power supply (T1 CP300 304p, from Iseg High Voltage, Radeberg, Germany) was used to apply voltage to the needle. A grounded cylindrical collector with 6 cm diameter was used to collect the fibers. Collector movements were controlled by motors with angular speeds between 3 and 4 rotations per minute. This set-up, depicted in Figure 1, allows the production of more uniform membranes. Additionally, the co-electrospinning of the PCL solution with the CS2 or with the CS4 solutions was performed onto the same collector by using two syringe pumps. The resulting membranes were designated PCS2 and PCS4, respectively. Table 1 displays the nomenclature of the scaffolds along with the composition of the solutions used to produce each of the scaffolds used in this work.

The parameters used to electrospin the 3 solutions are displayed in Table 2. These parameters were adjusted to achieve a steady electrospinning process, without jet interruptions nor solution accumulation at the needle tip. Fiber deposition proceeded for 10 h under controlled environmental conditions (relative humidity equal to 50 ± 5% and temperature equal to 20 ± 2 °C).

### 2.3. Characterization of Electrospun Membranes 

Scaffolds were kept in a desiccator under vacuum for at least two weeks before being used further to evaporate any residual solvent. To observe the morphology of the fibers, the electrospun membranes were sputter-coated with a mixture of gold/palladium and imaged with a TM 3030Plus Tabletop Scanning Electron Microscope from Hitachi (Tokyo, Japan) operating in high vacuum. The diameter of at least 100 fibers was measured for the PCL membrane using the ImageJ v1.50c4 software [31]. Results are expressed as the average ± experimental standard deviation.

The porosity, *P*, of the fibrous membranes was determined based on the apparent density method, using Equation (1):

(1) $$P=\frac{\rho_{0}-\rho }{\rho_{0}}\times 100$$

where *ρ* is the apparent density of the membranes (mass to volume ratio of the membranes) and *ρ*_0_ is the material density (PCL density or PCL/CS composite density). Samples were cut into 2.0 cm × 2.0 cm squares, their thickness was measured using a digital micrometer, and their mass was determined using an analytical balance (Radwag XA 82/220, Radom, Poland) to calculate their density. Twelve measurements were made using 3 different membranes of each composition. The results are presented as mean ± experimental standard deviation.

Mechanical properties were determined through uniaxial tensile stress–strain tests, using a Rheometric Scientific testing machine, loaded with a 20 N load cell at a crosshead speed of 2 mm/min at room temperature. The samples were cut with a rectangular shape, 3 cm × 1 cm, and their thickness was measured using a digital micrometer (Mitutoyo Corporation, Kawasaki, Japan). Young’s modulus was assessed from the slope of the linear region of the stress–strain curve, and the yield stress was obtained from the maximum stress before plastic deformation in the stress–strain curve. Twelve measurements were made using 3 different membranes of each composition. The results are presented as mean ± experimental standard deviation. Statistical significance was evaluated using ANOVA with Tukey Post Hoc test using the Kaleidagraph v5.06 software (Synergy Software, Reading, PA, USA).

### 2.4. Cell Culture

Human fetal foreskin fibroblasts (HFFF2 cell line, obtained from the ECACC, Porton Down, UK) at passage 13 were cultured in Dulbecco’s modified Eagle’s medium (complete DMEM, Sigma-Aldrich, St. Louis, Missouri, USA), supplemented with GlutaMAX, 10% *v*/*v* fetal bovine serum (FBS), 100 units/mL of penicillin, 100 µg/mL of streptomycin, and 2.50 µg/mL of amphotericin B, all from Life Technologies (Carlsbad, CA, USA). Fibroblasts were chosen for this study due to their physiological relevance, widespread presence in the human body, involvement in wound healing, and applications in tissue engineering.

Electrospun fiber membranes were cut with a 12 mm circle punch and sterilized with ethanol 70% during 30 min, followed by three washes with phosphate-buffered saline (PBS) solution and by soaking in complete culture medium. Membranes were held by home-made Teflon inserts placed inside a 24-well tissue culture plate. HFFF2 cells were seeded over a 0.5 cm^2^ area at a density of 10^4^ cells/cm^2^. The cells were seeded directly on the tissue culture plate and on glass coverslips as cell controls for the viability test or for fluorescence imaging, respectively. After seeding, the cells were incubated at 37 °C in a Sanyo MCO19AIC(UV) 5% CO_2_ humidified atmosphere incubator. 

Cell adhesion and proliferation were determined using a resazurin (Alfa Aesar, Ward Hill, MA, USA) solution (0.2 mg/mL in PBS) as a cell viability indicator. Briefly, the culture medium was replaced by complete medium supplemented with 50% of the resazurin solution. The cells were incubated for 3 h and then the absorbance was read at 570 nm and 600 nm using a microplate reader (Biotek ELX800UV, Winooski, VT, USA). The metabolic activity is assumed to be proportional to the corrected absorbance, which is obtained by subtracting the absorbance measured at 600 nm from the one measured at 570 nm. The combined standard uncertainty was calculated by propagation of uncertainties. The assay was performed on day 1 of culture to estimate cell adhesion, and then on days 8 and 14 of culture to assess cell proliferation. Analysis of variance (ANOVA) was performed using the OriginPro 2018 software from OriginLab (Northampton, MA, USA) to determine the significance of differences between samples. For multiple comparisons, Tukey’s test was used, and the differences were statistically significant at *p* < 0.05.

Fluorescent staining of the nucleus of cells growing on the different membranes was performed with 4′,6-diamidino-2-phenylindole (DAPI, from Thermo Fisher, Waltham, MA, USA) for day 1 cultures and with Helix NP™ Green (BioLegend, San Diego, CA, USA) for day 14 cultures. The cells were fixed with 3.7% paraformaldehyde, permeabilized with Triton X-100 (0.5% in phosphate-buffered saline, PBS), and stained with DAPI or Helix NP™ Green. The samples were mounted on glass coverslips with PBS and visualized with an epi-fluorescence microscope Nikon Eclipse Ti-S, equipped with a Nikon D610 digital camera. Both top and bottom surfaces of the scaffolds were imaged. Fluorescence images were processed using the software Fiji version 2.14.0/1.54 h [32]. The command Image: Color: Split Channels was applied to DAPI-stained images and the green channel selected for presentation. The command Process: Subtract Background (rolling ball radius of 1000 and Sliding paraboloid option) was applied to Helix NP™ Green-stained images.

## 3. Results

### 3.1. Fibrous Membrane Characterization

**Figure 2:**
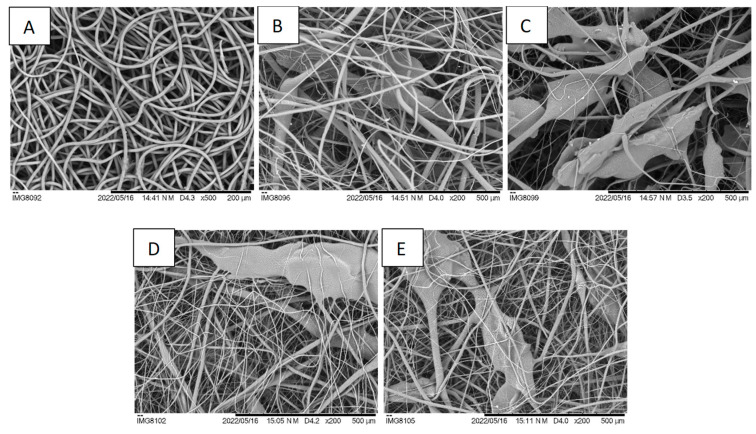
SEM images of (**A**) PCL, (**B**) CS2, (**C**) CS4, (**D**) PCS2, and (**E**) PCS4 electrospun nanofibers. The CS microgranules caused a significant expansion of the fibrous structure, with large pores appropriate for cell invasion. Scale bars: (**A**) 200 µm, (**B**–**E**) 500 µm.

SEM images of the different electrospun fiber membranes are shown in Figure 2. In PCL membranes, the fibers are randomly oriented, uniform, and defect-free, with minor variations in diameter along the fibers (Figure 2A). PCL fibers have a mean diameter of 3.4 ± 0.7 µm, similar to the results reported for PCL membranes prepared from PCL dissolved in chloroform but larger than those obtained when acetic acid is used as a solvent [33,34]. In PCL membranes with CS granules, fibers identical to those found on PCL-only membranes are interspersed with much larger ones, entrapping the CS granules, creating a non-uniform and rougher structure (Figure 2B,C). A higher content of CS granules is observed on CS4 membranes when compared to CS2 membranes, as expected. This structure also presents more scattered fibers, due to the presence of more CS granules which influence the formation of fibers with different diameters, as also reported by Arrieta et al. [35]. Co-electrospun PCL with CS2 or CS4 solutions resulted in membranes with a morphology in-between those of PCL and CS2 and CS4, with pores larger than those of the PCL membranes but not as large as those of the corresponding CS2 and CS4 membranes due to the presence of more PCL fibers (Figure 2D,E). The enlarged pores seen in CS-containing membranes are a requisite for cell migration across the scaffold [36]. 

The porosity of the membranes was determined using the method of apparent density. The fibrous membranes were all highly porous, with a porosity of approximately 88% (Table 3). The values obtained in this study are in excellent accordance with those reported by Liu et al. who used laser metrology to determine electrospun scaffold porosity [37]. There is no significant difference in porosity between the membranes with or without CS. This is somehow surprising since the SEM images reveal significantly different structural arrangements of the fibers. These results show that the random stacking of regular PCL fibers with irregular fibers and structures (the CS granules embedded in PCL) lead to scaffolds with the same porosity.

**Figure 3:**
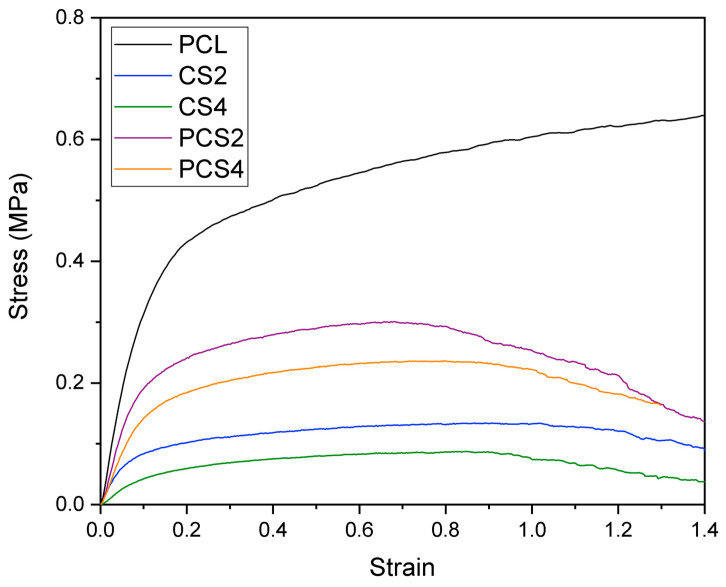
Typical stress–strain curves of electrospun membranes produced from PCL, PCL with CS granules (CS2 and CS4), or in co-deposition (PCS2 and PCS4).

The representative tensile stress–strain curves of each fibrous mat are depicted in Figure 3, and Young’s modulus and the yield stress are shown in Table 4. According to the literature, PCL electrospun membranes (produced using different solvents and concentrations) have a Young’s modulus between 2 and 10 MPa [38,39]. The Young’s modulus of PCL membranes was 5.5 ± 0.6 MPa, higher than the Young’s modulus of CS2, CS4, PCS2, and PCS4 membranes, with the differences between the Young’s modulus of PCL and the Young’s moduli of the other membranes all statistically significant (*p* < 0.001). A significant decrease of the Young’s modulus of PCL membranes with expanded pores was also observed by Hodge et al., despite their method involving the use of sacrificial PEO microparticles [40]. The incorporation of CS in the membrane disrupts the homogeneity of the PCL fibrous network causing the appearance of irregular fibers, some of which are very thin, while those that embed the CS granules are very large. Also, due to the dissimilar chemical nature of PCL and CS, electrostatic bonding is not favored. This causes a weakening of the PCL matrix due to the incorporation of the CS granules. 

When comparing the differences between Young’s moduli of membranes with different amounts of Cs granules (CS2 vs. CS4 and PCS2 vs. PCS4), although the nominal values are higher for those membranes with lower CS content, the differences are not statistically significant. Analyzing the effect of combining PCL fibers with the CS2 and CS4 fibers (CS2 vs. PCS2 and CS4 vs. PCS4), one concludes that the difference is statistically significant only in the case of CS4 vs. PCS4 (*p* < 0.001). Given that the CS4 membrane has the lowest Young’s modulus obtained in this work, 1.0 ± 0.6 MPa, blending these fibers with PCL fibers results in a significant improvement in the mechanical properties of the hybrid membrane. 

A similar behavior to Young’s modulus is seen for the yield stress: that of the PCL membrane is higher than that of any of the CS-containing membranes, with the differences between the yield stress of PCL and the yield stresses of the other membranes all statistically significant (*p* < 0.001). The yield stresses of the co-electrospun membranes are higher than those of the corresponding single membranes (PCS2 vs. CS2 and PCS4 vs. CS4), with the differences statistically significant, confirming the advantage, with respect to mechanical properties, of the co-electrospinning method.

### 3.2. In Vitro Evaluation of Membranes

**Figure 4:**
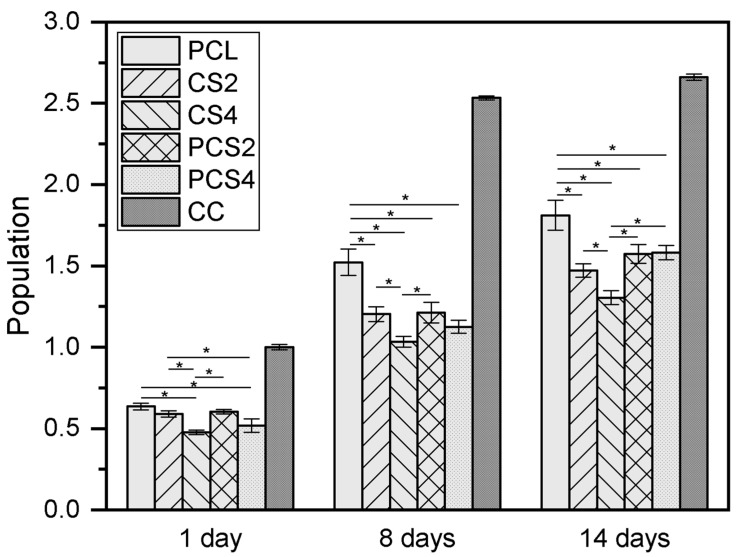
Population of HFFF2 cells cultured on the five different membranes and on tissue culture plastic (cell control, CC) on days 1, 8, and 14 of culture. Results are the mean ± standard deviation of at least three replicates from three independent assays. * ANOVA was performed to determine the significance of differences between samples; the differences were considered statistically significant at *p* < 0.05. The differences between CC and all other samples are statistically significant; they are not represented in the plot to avoid excessive clutter.

The cell adhesion and proliferation of HFFF2 cells seeded on electrospun scaffolds was evaluated using a resazurin colorimetric assay to evaluate cell population, which is assumed to be proportional to metabolic activity, over time. Results are depicted in Figure 4. Day 1 populations are interpreted as adhesion ratios, the fraction of cells seeded on each scaffold that succeeded in adhering to the scaffold and remained viable. The highest value was 64% of the cell control (cells cultured on tissue culture plastic) for the PCL scaffold. For the CS2 and CS4 scaffolds, the adhesion ratios were 59% and 48%, respectively, while for the PCS2 and PCS4 scaffolds, the adhesion ratios were 60% and 52%. After seeding, the establishment of a sufficient number of cell–scaffold adhesions must occur for the proper organization of the cytoskeleton and cell survival [41,42]. CS-containing scaffolds have a more open structure and cells have fewer anchorage sites for cell adhesion, leading to a lower survival. During the two weeks of culture, cells proliferated on all scaffolds, with the final populations being between 2.5 and 3.0 times the day 1 populations. On day 14, the cell population on the PCL scaffold was the highest and the differences statistically significant in comparison with all other scaffolds. The lowest cell population was that of the CS4 scaffold, with the differences statistically significant in comparison with all other scaffolds. Although the PCL scaffold was better at supporting cell proliferation, it did not support cell invasion, as presented below. 

**Figure 5:**
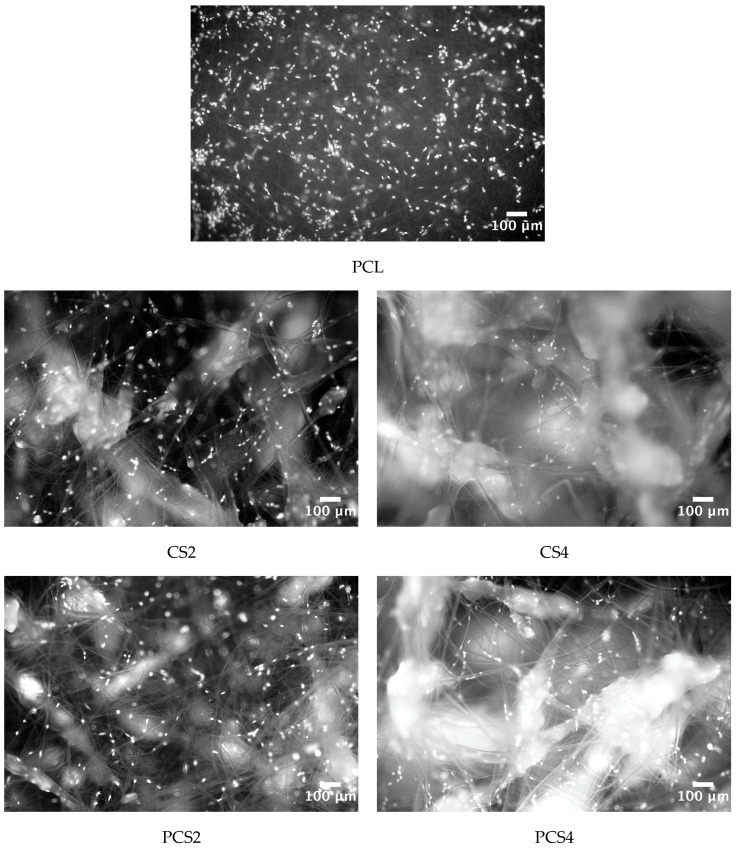
Nuclear staining with DAPI of HFFF2 cells seeded on the five scaffolds, 1 day after seeding. Chitosan’s auto-fluorescence when excited with UV light reveals the presence of the incorporated CS granules.

**Figure 6:**
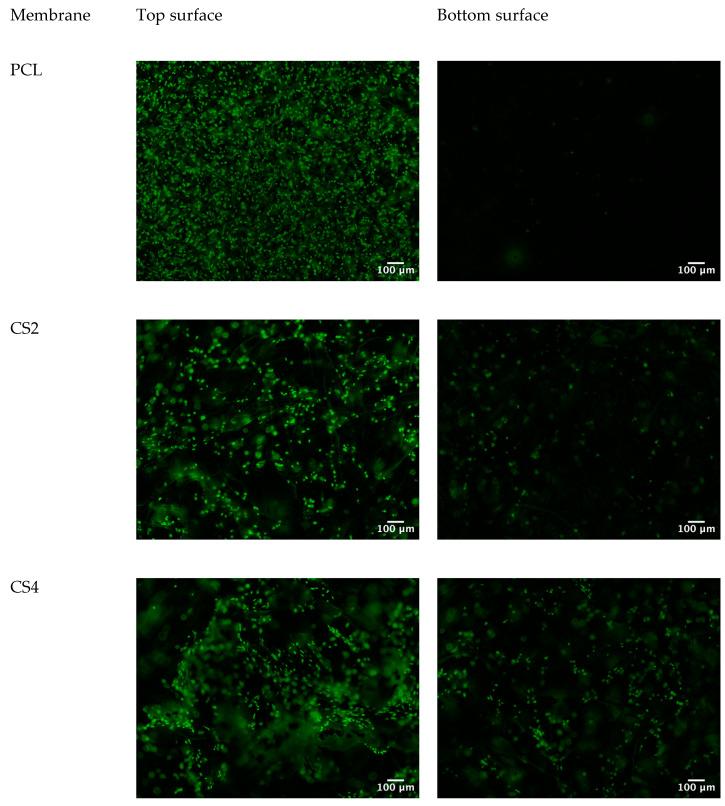

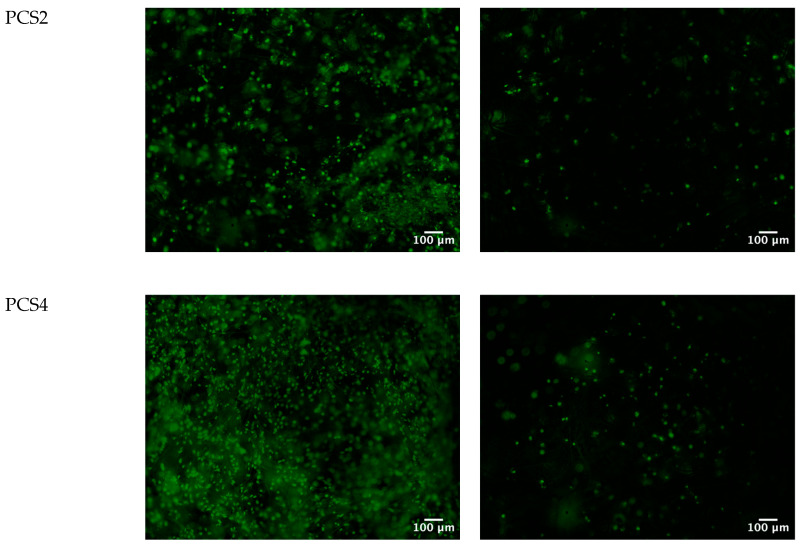
Nuclear staining using Helix NP Green of fibroblasts seeded on the five scaffolds, 14 days after seeding. An abundant population of cells is seen at the top surface of all scaffolds but only CS-containing scaffolds reveal the presence of cells at the bottom surface, revealing an effective cell infiltration into the scaffolds.

Electrospinning typically produces fibrous scaffolds with pores in the nanometer to a few micrometers range, insufficient for a true 3D cell culture scaffold [43]. Achieving pore sizes adequate for cell migration and scaffold vascularization requires modifications to the conventional electrospinning set-up or combinations with other techniques [14]. The strategy followed in the present work can be used with the typical electrospinning equipment and allows the production of scaffolds with an enlarged pore structure, as can be seen in Figure 2. The expanded pores created by the incorporation of CS granules are expected to promote cell infiltration. To test this hypothesis, nuclear staining of cells seeded on the five different scaffolds was performed on day 1 and on day 14 of culture. Day 1 results are shown in Figure 5. While on the PCL scaffold cells are at the surface or below just a few fibers, on the CS-containing scaffolds, the open structure reveals cells attached at the surface and deeper inside the scaffolds. On day 1, no cells could be detected at the bottom surface of the scaffolds. On day 14, cells could be detected both at the top surface and at the bottom surface of all scaffolds except for PCL (Figure 6). This shows that cells were able to infiltrate the scaffold and populate the whole scaffold’s volume, proving that the strategy investigated in this work is successful in expanding the structure of a fibrous scaffold produced using electrospinning so that it behaves as a true 3D structure capable of supporting cell adhesion, proliferation, and infiltration.

## 4. Discussion

Electrospinning produces packed arrangements of fibers resulting in membranes with high porosity but small pore size that prevent cellular infiltration [44]. To overcome this issue, different methods, including modifications of the electrospinning setup and sacrificial agents, have been used to create porous 3D structures that allow cell integration and vascularization [5]. Those methods usually require additional steps that increase production time and the cost of the procedure. 

In this work, using a single-step method, CS granules were incorporated in PCL membranes to expand the pores of the structure. The advantages of this approach lie in the fact that it does not require modifications to the conventional electrospinning set-up nor any post-processing steps. This approach can also be used with other combinations of polymers and particles, provided that the solvent used is a non-solvent for the material in granular form. Instead of PCL, other chloroform soluble polymers or copolymers can be used, such as poly(lactic acid), poly(glycolic acid), PLGA, or a polyhydroxyalkanoate.

The electrospinning processing conditions were optimized to produce PCL fibers, PCL fibers with CS granules, and hybrid membranes combining these two types of fiber. A similar approach was used by Valente et al., who studied the blending of PCL with CS in solution or in hybrid membranes and concluded that the same composition could lead to different cell responses according to the blending method used [26]. 

The CS-containing scaffolds have, indeed, as can be seen in Figure 2, a network of enlarged pores. Optimal pore size depends on each tissue, but cells and nutrients should have access to the interior of the scaffolds to create a three-dimensional structure similar to that of the ECM. For example, fibroblasts proliferate better in PCL membranes with a pore size of 6 to 20 µm [45].

The larger pores were achieved at the expense of a decrease in the mechanical properties of the membranes. Young’s modulus and the yield stress decreased with the presence of CS granules, particularly for the CS4 membrane, with higher CS content. Although this was expected, the co-electrospinning of CS2 or CS4 solutions with PCL resulted in hybrid membranes with mechanical integrity that preserved the expansion of the fibrous network suitable for cell infiltration. 

Research has shown that pore diameters should be at least the same magnitude as the dimensions of the cell to enhance cellular infiltration [46,47]. The new scaffolds produced allowed fibroblast infiltration compared to pure PCL fibers (Figure 5 and Figure 6). Avoiding the use of sacrificial agents ensures the structural integrity of the scaffold and the preservation of the expanded porous network. The new approach reported here allows the production of true 3D structures using the electrospinning technique that are able to support the adhesion, proliferation, and migration of fibroblasts throughout the volume of the scaffold. 

Other approaches to pore expansion using microparticles have been reported in the literature. Nam et al. introduced salt particles through a sheath surrounding the needle to produce a scaffold with a uniform but layered distribution of salt particles producing a partially delaminated structure [48]. Cells infiltrated the scaffold and migrated up to 4 mm after 3 weeks of culture. A similar layering effect was obtained by Jiang et al., who used a modified gas-foaming technique [49]. The expanded electrospun nanofiber scaffolds supported cellular infiltration and proliferation throughout the whole volume, whereas traditional nanofiber scaffolds displayed limited cellular proliferation on the surface. These methods allow adjustable gap widths and layer thicknesses by controlling processing parameters, but the layered structure causes a significant decrease in mechanical properties in the direction perpendicular to the layers.

The use of porogens to expands the network of electrospun fibers may prove difficult to achieve due to the partial collapse of the scaffold upon porogen leaching. Sprinkling poly(L-lactide) (PLLA) nanofibers with salt particles led to an increase in mean pore size from 5.5 µm for PLLA membranes to 48.7 µm for salt-sprinkled PLLA membranes after salt leaching, an average value much lower than might be expected since salt crystals were being deposited from a 710 µm mesh mandrel [50]. Still, MC3T3-E1 cells were able to infiltrate five times farther into the expanded PLLA scaffold after four weeks of culture. A significant decrease in mean pore size of non-woven PCL scaffolds after salt leaching in comparison to the original crystal size was also observed by Cortez Tornello et al. [51]. Since the microparticles used in the present work to expand the pores of the electrospun scaffolds do not need to be leached, dimensional shrinkage is not an issue with this method.

An interesting process to form porogens in situ is cryogenic electrospinning [52,53]. The collector is refrigerated, and the low temperature causes the ambient humidity to freeze concomitantly to fiber deposition. The ice crystals cause the expansion of the fibrous network and cells successfully infiltrate the scaffold [54]. An advantage of this method is that by adjusting polymer flow rate or ambient humidity, it is possible to change ice crystal and pore size during electrospinning. This is more difficult to achieve with the method used in the present work. An additional limitation of the current method is the amount of CS granules that can be incorporated in the solutions and still achieve successful electrospinning, which limits the expansion degree of the scaffolds. However, a higher CS granule concentration in the electrospinning solution does not seem to be necessary as 2 wt% already allowed for an effective cell invasion in CS2 and PCS2 scaffolds.

## 5. Conclusions

The goal of this study was to make it possible for cells to migrate across an electrospun scaffold. The strategy used consisted of creating expanded pores by incorporating CS granules into the PCL fibrous network. Analyzing the results globally, i.e., mechanical properties and cell adhesion, proliferation, and invasion, we conclude that the PCS2 membrane is the one that presents the best performance. Given the simplicity of the method and the successful achievement of cell infiltration along with the absence of significant mechanical weakening and dimensional shrinkage, this approach provides a new solution to overcome the limitations of flat membrane electrospinning and has great potential in tissue engineering applications. 

## Figures and Tables

**Table 1:** Nomenclature of the scaffolds and solutions used to produce them. The PCL concentration in solution is always 10 wt% whereas the CS concentration is either 2 or 4 wt%. The PCL, CS2, and CS4 membranes are produced using a simple electrospinning set-up. The PCS2 and PCS4 membranes are produced using a co-electrospinning set-up.

Name	Solution 1	Solution 2
PCL	PCL 10%	-
CS2	PCL 10% + CS 2%	-
CS4	PCL 10% + CS 4%	-
PCS2	PCL 10%	PCL 10% + CS 2%
PCS4	PCL 10%	PCL 10% + CS 4%

**Table 2:** Parameters used to electrospin the three solutions used in this work.

Solution	Flow Rate (mL/h)	Voltage (kV)	Distance (cm)
PCL	0.7	13	25
CS2	1.4	15	25
CS4	2.1	15	25

**Table 3:** Membrane thickness and density of bulk composite materials and porous electrospun membranes determined and used to calculate the porosity of electrospun fibrous membranes (mean ± experimental standard deviation).

Membrane	Thickness µm	Material Density g/cm^3^	Membrane Density g/cm^3^	Porosity %
PCL	227 ± 65	1.145	0.1354	88.2 ± 1.4
CS2	334 ± 28	1.136	0.1301	88.5 ± 2.0
CS4	756 ± 57	1.129	0.1223	89.1 ± 1.3
PCS2	346 ± 67	1.138	0.1403	87.7 ± 2.5
PCS4	381 ± 45	1.132	0.1414	87.5 ± 1.0

**Table 4:** Results of Young’s modulus (*Y*) and yield stress (σ_y_) of electrospun fibrous membranes. Columns labeled ANOVA indicate which pairs of values of *Y* and σ_y_ are statistically significant (with, at least, *p* < 0.005).

Membrane	*Y* (MPa)	*Y*, ANOVA	σ_y_ (MPa)	σ_y_, ANOVA
PCL	5.5 ± 0.6	CS2, CS4, PCS2, PCS4	0.46 ± 0.09	CS2, CS4, PCS2, PCS4
CS2	2.0 ± 1.0	PCL	0.13 ± 0.02	PCL, PCS2
CS4	1.0 ± 0.6	PCL, PCS4	0.08 ± 0.02	PCL, PCS2, PCS4
PCS2	2.6 ± 1.4	PCL	0.23 ± 0.07	PCL, CS2, CS4
PCS4	2.2 ± 0.9	PCL, CS4	0.18 ± 0.04	PCL, CS4

## Data Availability

Dataset available on request from the authors.

## References

[1] Vig K., Chaudhari A., Tripathi S., Dixit S., Sahu R., Pillai S., Dennis V.A., Singh S.R. (2017). Advances in Skin Regeneration Using Tissue Engineering. Int. J. Mol. Sci..

[2] Chen J., Fan Y., Dong G., Zhou H., Du R., Tang X., Ying Y., Li J. (2023). Designing Biomimetic Scaffolds for Skin Tissue Engineering. Biomater. Sci..

[3] Fromager B., Marhuenda E., Louis B., Bakalara N., Cambedouzou J., Cornu D. (2023). Recent Advances in Electrospun Fibers for Biological Applications. Macromol.

[4] Rnjak-Kovacina J., Weiss A.S. (2011). Increasing the Pore Size of Electrospun Scaffolds. Tissue Eng. Part. B Rev..

[5] Han S., Nie K., Li J., Sun Q., Wang X., Li X., Li Q. (2021). 3D Electrospun Nanofiber-Based Scaffolds: From Preparations and Properties to Tissue Regeneration Applications. Stem Cells Int..

[6] Mandal B.B., Kundu S.C. (2009). Cell Proliferation and Migration in Silk Fibroin 3D Scaffolds. Biomaterials.

[7] Akay G., Birch M.A., Bokhari M.A. (2004). Microcellular polyHIPE Polymer Supports Osteoblast Growth and Bone Formation in Vitro. Biomaterials.

[8] Zhang Z., Feng Y., Wang L., Liu D., Qin C., Shi Y. (2022). A Review of Preparation Methods of Porous Skin Tissue Engineering Scaffolds. Mater. Today Commun..

[9] Wu J., Hong Y. (2016). Enhancing Cell Infiltration of Electrospun Fibrous Scaffolds in Tissue Regeneration. Bioact. Mater..

[10] Wang Z., Cui Y., Wang J., Yang X., Wu Y., Wang K., Gao X., Li D., Li Y., Zheng X.-L.L. (2014). The Effect of Thick Fibers and Large Pores of Electrospun Poly(ε-Caprolactone) Vascular Grafts on Macrophage Polarization and Arterial Regeneration. Biomaterials.

[11] Jiang J., Li Z., Wang H., Wang Y., Carlson M.A., Teusink M.J., MacEwan M.R., Gu L., Xie J. (2016). Expanded 3D Nanofiber Scaffolds: Cell Penetration, Neovascularization, and Host Response. Adv. Healthc. Mater..

[12] Ameer P.R. (2019). Kasoju Strategies to Tune Electrospun Scaffold Porosity for Effective Cell Response in Tissue Engineering. J. Funct. Biomater..

[13] Zhong S., Zhang Y., Lim C.T. (2012). Fabrication of Large Pores in Electrospun Nanofibrous Scaffolds for Cellular Infiltration: A Review. Tissue Eng. Part. B Rev..

[14] Feltz K.P., Growney Kalaf E.A., Chen C., Martin R.S., Sell S.A. (2017). A Review of Electrospinning Manipulation Techniques to Direct Fiber Deposition and Maximize Pore Size. Electrospinning.

[15] Jun I., Han H.-S., Edwards J., Jeon H. (2018). Electrospun Fibrous Scaffolds for Tissue Engineering: Viewpoints on Architecture and Fabrication. Int. J. Mol. Sci..

[16] Bongiovanni Abel S., Montini Ballarin F., Abraham G.A. (2020). Combination of Electrospinning with Other Techniques for the Fabrication of 3D Polymeric and Composite Nanofibrous Scaffolds with Improved Cellular Interactions. Nanotechnology.

[17] Semitela Â., Girão A.F., Fernandes C., Ramalho G., Bdikin I., Completo A., Marques P.A. (2020). Electrospinning of Bioactive Polycaprolactone-Gelatin Nanofibres with Increased Pore Size for Cartilage Tissue Engineering Applications. J. Biomater. Appl..

[18] Sadeghi-avalshahr A.R., Nokhasteh S., Molavi A.M., Mohammad-pour N., Sadeghi M. (2020). Tailored PCL Scaffolds as Skin Substitutes Using Sacrificial PVP Fibers and Collagen/Chitosan Blends. Int. J. Mol. Sci..

[19] Ekaputra A.K., Prestwich G.D., Cool S.M., Hutmacher D.W. (2008). Combining Electrospun Scaffolds with Electrosprayed Hydrogels Leads to Three-Dimensional Cellularization of Hybrid Constructs. Biomacromolecules.

[20] Hodge J.G., Quint C. (2022). Improved Porosity of Electrospun Poly (Lactic-Co-Glycolic) Scaffolds by Sacrificial Microparticles Enhances Cellular Infiltration Compared to Sacrificial Microfiber. J. Biomater. Appl..

[21] Zander N.E., Orlicki J.A., Rawlett A.M., Beebe T.P. (2013). Electrospun Polycaprolactone Scaffolds with Tailored Porosity Using Two Approaches for Enhanced Cellular Infiltration. J. Mater. Sci. Mater. Med..

[22] Singh G., Chanda A. (2021). Mechanical Properties of Whole-Body Soft Human Tissues: A Review. Biomed. Mater..

[23] Gomes S., Rodrigues G., Martins G., Henriques C., Silva J.C. (2017). Evaluation of Nanofibrous Scaffolds Obtained from Blends of Chitosan, Gelatin and Polycaprolactone for Skin Tissue Engineering. Int. J. Biol. Macromol..

[24] Kou S.G., Peters L., Mucalo M. (2022). Chitosan: A Review of Molecular Structure, Bioactivities and Interactions with the Human Body and Micro-Organisms. Carbohydr. Polym..

[25] Abourehab M.A.S., Pramanik S., Abdelgawad M.A., Abualsoud B.M., Kadi A., Ansari M.J., Deepak A. (2022). Recent Advances of Chitosan Formulations in Biomedical Applications. Int. J. Mol. Sci..

[26] Valente T., Ferreira J.L., Henriques C., Borges J.P., Silva J.C. (2019). Polymer Blending or Fiber Blending: A Comparative Study Using Chitosan and Poly(ε-Caprolactone) Electrospun Fibers. J. Appl. Polym. Sci..

[27] Jung S.-M., Yoon G.H., Lee H.C., Shin H.S. (2015). Chitosan Nanoparticle/PCL Nanofiber Composite for Wound Dressing and Drug Delivery. J. Biomater. Sci. Polym. Ed..

[28] Wang S., Li Y., Zhao R., Jin T., Zhang L., Li X. (2017). Chitosan Surface Modified Electrospun Poly(ε-Caprolactone)/Carbon Nanotube Composite Fibers with Enhanced Mechanical, Cell Proliferation and Antibacterial Properties. Int. J. Biol. Macromol..

[29] Tardajos M.G., Cama G., Dash M., Misseeuw L., Gheysens T., Gorzelanny C., Coenye T., Dubruel P. (2018). Chitosan Functionalized Poly-ε-Caprolactone Electrospun Fibers and 3D Printed Scaffolds as Antibacterial Materials for Tissue Engineering Applications. Carbohydr. Polym..

[30] Querido D., Vieira T., Ferreira J.L., Henriques C., Borges J.P., Silva J.C. (2022). Study on the Incorporation of Chitosan Flakes in Electrospun Polycaprolactone Scaffolds. Polymers.

[31] Schneider C.A., Rasband W.S., Eliceiri K.W. (2012). NIH Image to ImageJ: 25 Years of Image Analysis. Nat. Methods.

[32] Schindelin J., Arganda-Carreras I., Frise E., Kaynig V., Longair M., Pietzsch T., Preibisch S., Rueden C., Saalfeld S., Schmid B. (2012). Fiji: An Open-Source Platform for Biological-Image Analysis. Nat. Methods.

[33] Shin M., Yoshimoto H., Vacanti J.P. (2004). In Vivo Bone Tissue Engineering Using Mesenchymal Stem Cells on a Novel Electrospun Nanofibrous Scaffold. Tissue Eng..

[34] Ferreira J.L., Gomes S., Henriques C., Borges J.P., Silva J.C. (2014). Electrospinning Polycaprolactone Dissolved in Glacial Acetic Acid: Fiber Production, Nonwoven Characterization, and In Vitro Evaluation. J. Appl. Polym. Sci..

[35] Arrieta M.P., López J., López D., Kenny J.M., Peponi L. (2016). Effect of Chitosan and Catechin Addition on the Structural, Thermal, Mechanical and Disintegration Properties of Plasticized Electrospun PLA-PHB Biocomposites. Polym. Degrad. Stab..

[36] Bružauskaitė I., Bironaitė D., Bagdonas E., Bernotienė E. (2016). Scaffolds and Cells for Tissue Regeneration: Different Scaffold Pore Sizes—Different Cell Effects. Cytotechnology.

[37] Liu Y., Chaparro F.J., Tian Z., Jia Y., Gosser J., Gaumer J., Ross L., Tafreshi H., Lannutti J.J. (2023). Visualization of Porosity and Pore Size Gradients in Electrospun Scaffolds Using Laser Metrology. PLoS ONE.

[38] Croisier F., Duwez A.-S.S., Jérôme C., Léonard A.F.F., Van Der Werf K.O.O., Dijkstra P.J.J., Bennink M.L.L. (2012). Mechanical Testing of Electrospun PCL Fibers. Acta Biomater..

[39] Gomes S., Querido D., Ferreira J.L., Borges J.P., Henriques C., Silva J.C. (2019). Using Water to Control Electrospun Polycaprolactone Fibre Morphology for Soft Tissue Engineering. J. Polym. Res..

[40] Hodge J., Quint C. (2019). The Improvement of Cell Infiltration in an Electrospun Scaffold with Multiple Synthetic Biodegradable Polymers Using Sacrificial PEO Microparticles. J. Biomed. Mater. Res. A.

[41] Khalili A., Ahmad M. (2015). A Review of Cell Adhesion Studies for Biomedical and Biological Applications. Int. J. Mol. Sci..

[42] Wang F., Cai X., Shen Y., Meng L. (2023). Cell–Scaffold Interactions in Tissue Engineering for Oral and Craniofacial Reconstruction. Bioact. Mater..

[43] Zhang Y., Zhang M., Cheng D., Xu S., Du C., Xie L., Zhao W. (2022). Applications of Electrospun Scaffolds with Enlarged Pores in Tissue Engineering. Biomater. Sci..

[44] Zulkifli M.Z.A., Nordin D., Shaari N., Kamarudin S.K. (2023). Overview of Electrospinning for Tissue Engineering Applications. Polymers.

[45] Lowery J.L., Datta N., Rutledge G.C. (2010). Effect of Fiber Diameter, Pore Size and Seeding Method on Growth of Human Dermal Fibroblasts in Electrospun Poly(ɛ-Caprolactone) Fibrous Mats. Biomaterials.

[46] Balguid A., Mol A., van Marion M.H., Bank R.A., Bouten C.V.C.C., Baaijens F.P.T.T. (2009). Tailoring Fiber Diameter in Electrospun Poly(ɛ-Caprolactone) Scaffolds for Optimal Cellular Infiltration in Cardiovascular Tissue Engineering. Tissue Eng. Part. A.

[47] Whited B.M., Whitney J.R., Hofmann M.C., Xu Y., Rylander M.N. (2011). Pre-Osteoblast Infiltration and Differentiation in Highly Porous Apatite-Coated PLLA Electrospun Scaffolds. Biomaterials.

[48] Nam J., Huang Y., Agarwal S., Lannutti J. (2007). Improved Cellular Infiltration in Electrospun Fiber via Engineered Porosity. Tissue Eng..

[49] Jiang J., Carlson M.A., Teusink M.J., Wang H., MacEwan M.R., Xie J. (2015). Expanding Two-Dimensional Electrospun Nanofiber Membranes in the Third Dimension by a Modified Gas-Foaming Technique. ACS Biomater. Sci. Eng..

[50] Wright L.D., Andric T., Freeman J.W. (2011). Utilizing NaCl to Increase the Porosity of Electrospun Materials. Mater. Sci. Eng. C.

[51] Cortez Tornello P.R., Caracciolo P.C., Igartúa Roselló J.I., Abraham G.A. (2018). Electrospun Scaffolds with Enlarged Pore Size: Porosimetry Analysis. Mater. Lett..

[52] Simonet M., Schneider O.D., Neuenschwander P., Stark W.J. (2007). Ultraporous 3D Polymer Meshes by Low-Temperature Electrospinning: Use of Ice Crystals as a Removable Void Template. Polym. Eng. Sci..

[53] Leong M.F., Chan W.Y., Chian K.S. (2013). Cryogenic Electrospinning: Proposed Mechanism, Process Parameters and Its Use in Engineering of Bilayered Tissue Structures. Nanomedicine.

[54] Leong M.F., Rasheed M.Z., Lim T.C., Chian K.S. (2009). In Vitro Cell Infiltration and *in Vivo* Cell Infiltration and Vascularization in a Fibrous, Highly Porous Poly(D,L-lactide) Scaffold Fabricated by Cryogenic Electrospinning Technique. J. Biomed. Mater. Res. A.

